# Shock From Twisting Peaks: A Rare Case of Recurrent Torsades de Pointes Secondary to Leuprolide-Induced Prolonged QT

**DOI:** 10.7759/cureus.9041

**Published:** 2020-07-07

**Authors:** Danish Abbasi, Saif Faiek, Sanjay Shetty, Ejaz Khan

**Affiliations:** 1 Cardiovascular Diseases, University of Arkansas, Little Rock, USA; 2 Internal Medicine, AtlantiCare Regional Medical Center, Atlantic City, USA; 3 Cardiovascular Medicine, AtlantiCare Regional Medical Center, Atlantic City, USA; 4 Cardiac Electrophysiology, Atlanticare Regional Medical Center, Atlantic City, USA

**Keywords:** leuprolide, torsades de pointes (tdp), leuprolide-induced arrhythmia, leuprolide-induced torsades de pointes, drug-induced torsades de pointes

## Abstract

Leuprolide acetate is a synthetic nonpeptide analog that is a potent gonadotropin-releasing hormone receptor agonist. It is used in diverse clinical applications, including treatment for prostate cancer, endometriosis, and uterine fibroids as well as the in vitro fertilization technique. Prolonged QT interval leading to torsades de pointes (TdP) is one of the very rare side effects of leuprolide therapy. Herein, we report a 68-year-old male patient with a history of prostate cancer post-radiation and on androgen suppression therapy with leuprolide who suffered from out-of-hospital cardiac arrest. After initial resuscitation, the patient’s electrocardiogram (ECG) showed a prolonged corrected QT interval (QTc), which subsequently progressed into a TdP rhythm, requiring lidocaine drip initially. The patient’s symptoms improved, and his ECG rhythm was resolved after initiating mexiletine and propranolol treatment with no recurrent TdP episodes after discontinuation of leuprolide.

## Introduction

Torsades de pointes (TdP) arrhythmia is a potentially life-threatening clinical condition that occurs as a complication of a prolonged QT interval [1]. Leuprolide has been associated with the risk of QT prolongation in clinical trials; however, to our knowledge, no clinical cases have been reported. We believe this is the first reported case of recurrent TdP in a patient receiving leuprolide treatment.

## Case presentation

**Figure 1:**
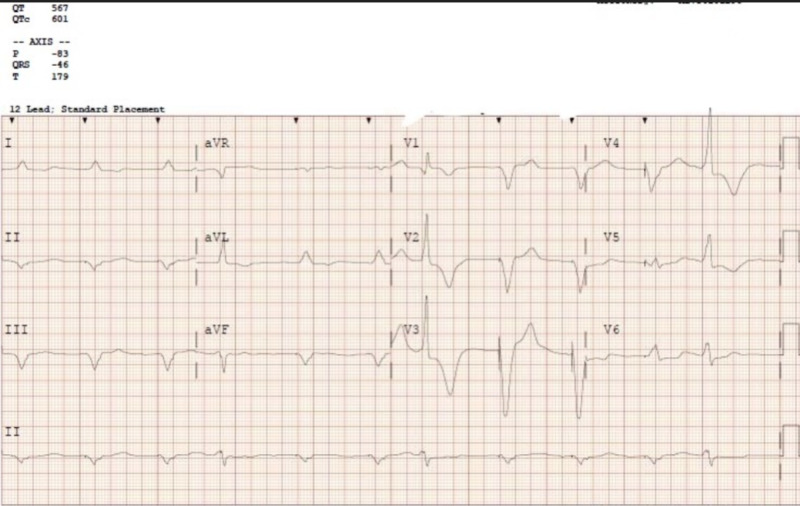
Electrocardiogram on presentation showing prolonged QT interval

A 68-year-old male with a past medical history of prostate cancer post-radiation, on androgen suppression therapy, hypertension, dyslipidemia, St. Jude aortic valve (St. Jude Medical, Inc., St. Paul, MN) replacement in 2009, heart failure, and atrial fibrillation status post-Medtronic pacemaker (Medtronic plc, Minneapolis, MN) placement in 2014 was seen in the hospital after suffering from out-of-hospital cardiac arrest. The patient was in a casino with his wife when she noticed that he did not appear well; subsequently, he became unconscious. He was found to have pulseless electrical activity by the first responders. Chest compressions were initiated, and he received two rounds of epinephrine, after which he was successfully resuscitated. The patient was intubated in the field and transferred to the hospital. His initial blood laboratory workup showed the following values: blood sugar 238 mg/dL, blood urea nitrogen (BUN) 25, creatinine (Cr) 1.27 mg/dL, glomerular filtration rate (GFR) 56 mL/min, sodium 143 mmol/mL, potassium 3.8 mmol/mL, chloride 110 mmol/mL, bicarbonate 21 mmol/mL, calcium 8.5 mg/dL, magnesium 2.0 mg/dL, creatine phosphokinase (CPK) 641 U/L, creatine kinase MB (CK-MB) 18.38 ng/mL, and troponin 0.240 ng/mL. The ECG showed a ventricular paced rhythm with a prolonged QT interval. The corrected QT interval (QTc) on presentation was 601 ms (Figure 1). 

**Figure 2:**
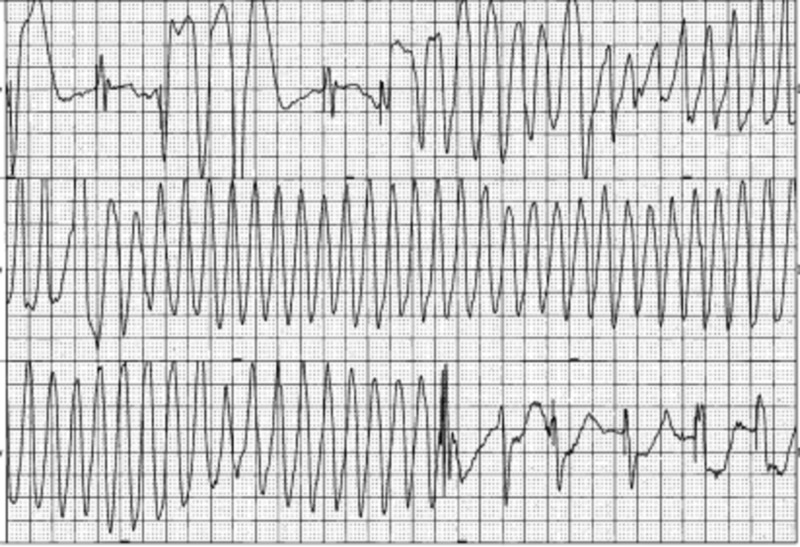
Polymorphic ventricular tachycardia

**Figure 3:**
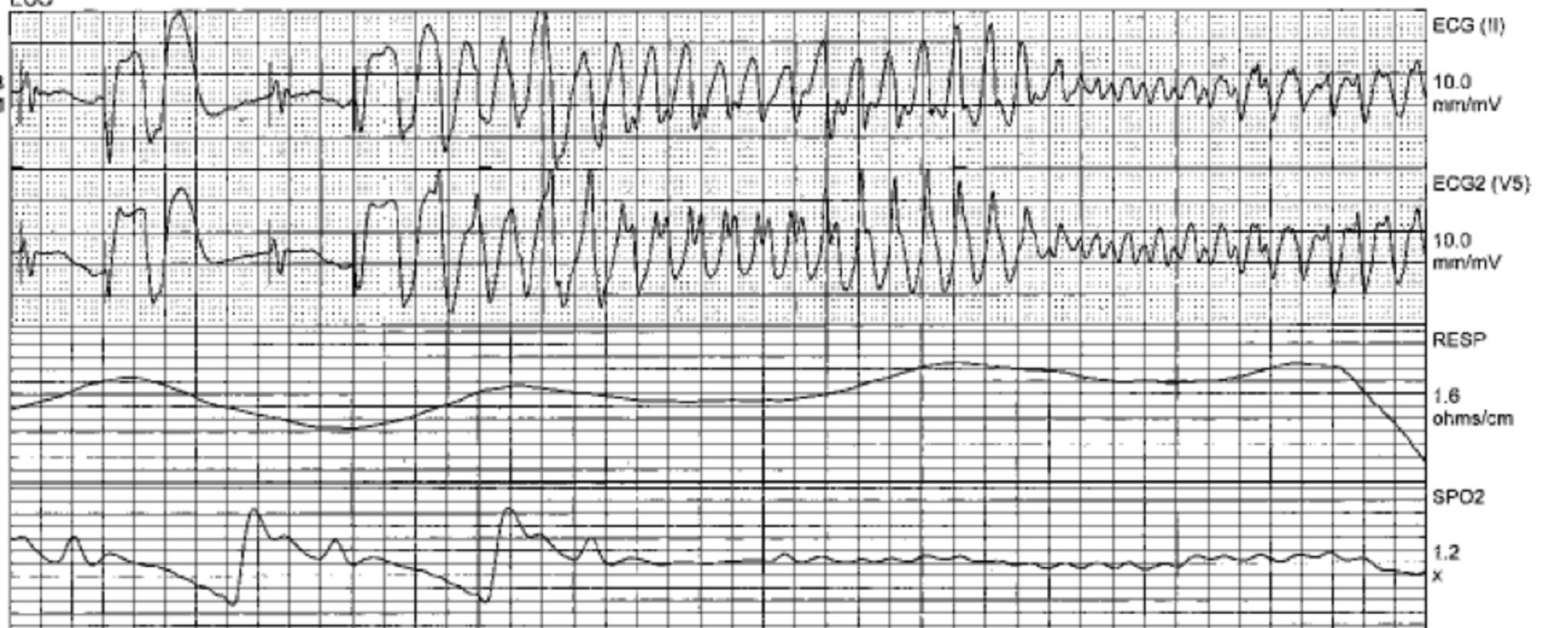
Polymorphic ventricular tachycardia and torsades de pointes

The patient was following simple commands off sedation, and therefore, the hypothermia protocol was not initiated. The patient was extubated overnight. He began suffering from multiple runs of sustained ventricular tachycardia (Figure 2) and was started on amiodarone initially. The rhythm progressed to TdP (Figure 3), and a lidocaine drip was started immediately. He received magnesium sulfate, and the cardiac electrophysiologist was consulted. The patient exhibited an ejection fraction of 40% on echo and was 100% right ventricular paced. Cardiac catheterization did not show significant coronary artery disease. The patient’s pacemaker was upgraded to a biventricular implantable cardioverter defibrillator. Thereafter, he suffered from another episode of TdP and was shocked with his implantable cardioverter defibrillator. The lidocaine drip was discontinued, and he was started on mexiletine and propranolol. The propranolol dose was increased to 40 mg BID with subsequent improvement in the rhythm. He was restarted on coumadin as per his preference and was discharged from the hospital with instructions to follow up with his cardiologist as an outpatient.

**Figure 4:**
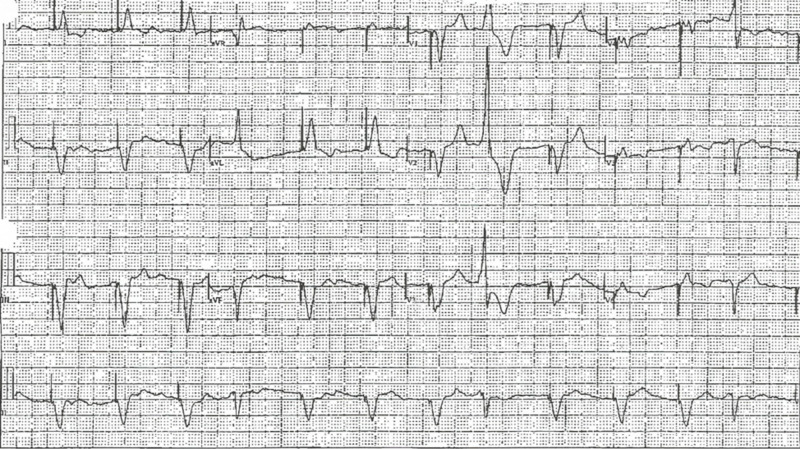
Electrocardiogram showing prolonged QT interval

The patient was diagnosed with prostate cancer in June 2015. He had Gleason 8, T2a N0 prostate adenocarcinoma for which he declined any surgical intervention. He received radiation therapy along with adjunctive androgen suppression therapy. The patient was treated with lupron (leuprolide) depot (30 mg every four months). His ECG three months prior to the event showed a ventricular paced rhythm with a QTc of 498 ms and an ECG QSR duration of 177 ms (Figure 4). The patient’s medications included flomax (tamsulosin; 0.4 mg), coumadin (7.5 mg), lipitor (10 mg), valsartan/hydrochlorothiazide (160 mg/12.5 mg), and lupron (leuprolide; 30 mg). He did not have any family history of long QT syndrome (LQTS) and had not previously suffered from ventricular tachycardia events.

**Figure 5:**
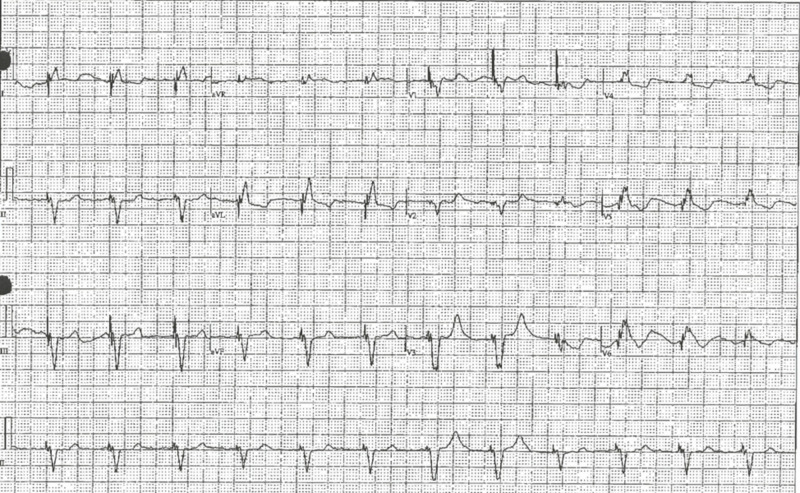
Follow-up electrocardiogram showing improved QT interval

After an extensive review of the patient’s medications, his prolonged QT was attributed to the rare side effect of androgen suppression therapy with lupron (leuprolide). The patient did not receive any further treatment with lupron (leuprolide) after this event. In the six-month follow-up visit, the patient revealed that he had not suffered from any further episodes of ventricular tachycardia or ventricular fibrillation. Moreover, his ECG showed an improvement in the QTc duration, which had reduced to 484 ms (Figure 5). The patient continued follow-up with his outpatient cardiologist. 

## Discussion

In 1966, Francois Dessertenne described a specific electrocardiographic form of polymorphic ventricular tachycardia, which he termed “torsades de pointes” (translated as “twisting of peaks”). TdP is a potentially lethal ventricular dysrhythmia resulting from a complication of a prolonged QT interval [1]. It is difficult to measure TdP mortality, as an ECG showing TdP arrhythmia may not be available during sudden cardiac death. However, many studies have found that drugs that prolong the QT interval are associated with a significantly high risk of sudden cardiac death [2]. Although the number of medications related to TdP incidence has increased in recent years, cell-based assays for the prediction of a drug’s potential to induce TdP in humans are still lacking. Drugs that prolong action potential duration, cause ectopic beats, and increase the dispersion of ventricular repolarization are more likely to cause TdP. However, no exact relationship between these changes and the development of TdP has been established [1,3].

Medications prescribed for noncardiac conditions can lead to QT prolongation and might trigger TdP. These include nonsedating antihistamines, antibiotics, antipsychotics, antidepressants, and gastrointestinal prokinetic drugs [1]. Zeltser et al. reviewed 249 cases of TdP secondary to noncardiac drug use. They created a list of “easily identifiable risk factors”, including (1) female gender; (2) heart disease, including myocardial infarction, heart failure, valvular disease, or cardiomyopathy; (3) hypokalemia; (4) drug toxicity not secondary to suicide attempts but identified as the administration of doses above recommended dosages or administration of standard treatments to patients with impaired drug metabolism; (5) drug interactions, defined as the administration of two or more drugs that prolong the QT interval; and (6) a history of familial LQTS, drug-induced torsades, or a baseline prolonged QT interval (QTc: 450 ms) on the ECG. According to the analysis, the most common risk factor was female gender (71%). Almost all patients had at least one risk factor, while 71% had two or more risk factors [4]. A population-based case-control study in the Netherlands conducted by Straus et al. on noncardiac drugs that prolong the QT interval found an approximately threefold higher risk of death (95% confidence interval: 1.6 to 4.7) among patients who had been treated with these medications. The study estimated that noncardiac drugs cause > 15,000 deaths annually in the United States and Europe [5]. 

The European Heart Journal published a transatlantic clinical approach to diagnosis and therapy for LQTS [6]. They recommended a diagnostic criterion based on ECG findings, clinical history, and family history. Patients with a score of ≤1 point have a low probability of developing LQTS, whereas those scoring 1.5 to 3 points are said to have an intermediate probability of developing the syndrome. Patients with a score of ≥3.5 points have a high probability of LQTS diagnosis. Our patient had a score of 6, thus indicating a high probability for the syndrome’s development.

The management of TdP is based on the patient’s clinical stability. An acute episode of TdP causing hemodynamic compromise is treated with unsynchronized cardioversion. Medical management includes correction of electrolyte imbalance, removal of the culprit drug, intensive cardiac monitoring, and immediate administration of intravenous magnesium sulfate. In cases of drug-induced LQTS or TdP, the cessation of the offending drug(s) is essential. However, until the drug clearance is complete, patients with frequent long runs of TdP will require treatment to shorten the QT interval.

β-adrenergic blocking agents are the first-choice therapy for symptomatic LQTS patients. The efficacy of β-blockers was analyzed by Moss et al. in 869 LQTS patients [7]. Patients on β-blockers showed a significant reduction in the rate of cardiac events. However, symptomatic patients were still at risk of recurrent cardiac complications while on therapy. Not all β-blockers are equally effective. Chockalingam et al. compared propranolol, metoprolol, and nadolol in 382 patients. Symptoms before therapy and the first breakthrough cardiac events were documented. The study showed the superiority of propranolol as compared to metoprolol in QTc shortening. Symptomatic patients receiving propranolol/nadolol also had a higher event-free survival compared to those being treated with metoprolol [8]. 

Prostate cancer is the second most common cancer in men worldwide. According to the American Cancer Society, the overall incidence of cancer decreased by 3.1% per year from 2009 to 2012. Androgen deprivation therapy or androgen suppression therapy is an essential aspect in the treatment of advanced prostate cancer. Leuprolide acetate is a synthetic nonpeptide analog and a potent gonadotropin-releasing hormone receptor agonist. It is used for diverse clinical applications, including treatment of prostate cancer, endometriosis, and uterine fibroids as well as in vitro fertilization techniques. Leuprolide acetate suppresses the secretion of luteinizing hormone and follicle-stimulating hormone, which subsequently curbs gonadal sex steroid production [9]. Androgen therapy in conjunction with radiation therapy improves survival in prostate cancer patients [10]. A review by Saylor and Smith showed that androgen deprivation therapy increases obesity, decreases insulin sensitivity, and adversely alters lipid profiles. It may be associated with a higher incidence of diabetes and cardiovascular disease [11].

The association between leuprolide and QT prolongation has been reported in clinical trials only. Smith et al. compared cardiovascular adverse events between leuprolide acetate and degarelix in a one-year randomized phase III trial and found markedly abnormal QTc values (500 ms or higher) in only 1% of the leuprolide group. Supraventricular arrhythmias were the most common type of arrhythmia, affecting 4% of the leuprolide group. The most frequently reported cardiac disorder was ischemic heart disease, which occurred in 10% of those on leuprolide [12]. Three randomized phase III studies were conducted to compare the gonadotropin-releasing hormone antagonist abarelix with goserelin plus bicalutamide (G + B) (study 1), leuprolide (L) monotherapy (study 2), or leuprolide plus bicalutamide (L + B) (study 3) in patients with prostate cancer. The Bazett and Fredericia QT intervals were measured at the baseline. Hormonal therapy, which induced androgen deficiency, was associated with prolongation of the QT interval [13].

## Conclusions

Recurrent episodes of TdP are associated with high morbidity and mortality. Androgen suppression therapy has been shown to prolong the QT interval; however, cases of clinically significant ventricular arrhythmias are rare. We present a case of cardiac arrest due to recurrent TdP secondary to leuprolide therapy in a patient with prostate cancer.
